# Treatment Reproducibility in Brain Stereotactic Radiotherapy Using a Shim Mask Versus a Standard Mask

**DOI:** 10.7759/cureus.66108

**Published:** 2024-08-04

**Authors:** Zaheeda Mulla, Rania Hashem, Victor Joseph, Hani Maumenah, Amina Weber, Abdulhameed Khasim, Huda Altoukhi

**Affiliations:** 1 Oncology, King Faisal Specialist Hospital and Research Center, Jeddah, SAU; 2 Radiology, King Abdul-Aziz University, Jeddah, SAU; 3 Radiology, King Abdul-Aziz University Hospital, Jeddah, SAU

**Keywords:** cone-beam computed tomography (cbct), stereotactic radiotherapy (srt), stereotactic radiosurgery (srs), immobilization accuracy, brain tumor

## Abstract

Introduction

This study aimed to evaluate the setup accuracy of the new shim mask with mouth bite compared to the standard full brain mask in stereotactic radiosurgery (SRS) and radiotherapy (SRT) treatments for brain metastases or tumors.

Method

A combined retrospective and prospective design was employed, involving 40 patients treated at our center. Patients previously treated using standard head masks formed the retrospective cohort, while those treated with the Shim mask and mouth bite formed the prospective cohort. Daily cone-beam computed tomography (CBCT) scans were obtained before each treatment session to ensure patient setup accuracy. Key metrics included absolute shifts in translational and rotational directions, the number of repeat CBCTs, and the time interval between CBCTs.

Results

The Shim mask significantly reduced the mean setup errors in the lateral translation (p=0.022) from 0.17 cm (SD=0.10) to 0.10 cm (SD=0.10), and in X-axis rotation (p=0.030) from 0.79° (SD=0.43) to 0.47° (SD=0.47). By considering cutoff points of 1 mm in translational and 1° in rotational directions, the Shim mask was significantly more accurate in the lateral direction (p=0.004). Moreover, while 70% of patients in the standard group required repeat CBCT scans, none in the Shim group did, resulting in an average time saving of 10.4 minutes per patient.

Conclusion

The Shim mask with mouth bite offers enhanced immobilization accuracy in SRT/SRS treatments, leading to time and potential cost savings by reducing the need for repeat CBCT scans. This underscores the importance of adopting innovative immobilization techniques to optimize patient outcomes.

## Introduction

The treatment of brain metastases has significantly advanced with the introduction of linac1-based stereotactic radiosurgery (SRS) and radiotherapy (SRT) techniques [1-3]. SRS involves a single, substantial radiation dose, while SRT delivers several high doses to a precisely defined target. Key to these methods is the ability to create sharp dose gradients that effectively spare the surrounding healthy tissues [2-4]. Accurate positioning and immobilization are essential due to the high doses and precision required [5]. Traditionally, our unit's standard care for immobilizing patients undergoing brain SRT involves a full-head thermoplastic mask. However, an emerging alternative is the innovative Integrated Shim™ technology developed by CQ Medical™, formerly Qfix®. The Integrated Shim™ technology is a Shim head mask with an integrated mouth bite, designed to enhance setup accuracy and potentially reduce setup errors [2]. What makes it different from the standard mask is that it allows independent height adjustment at each pin. Studies have shown that immobilization devices can cause significant anxiety among patients, with many finding the mask claustrophobic and requesting modifications for comfort, pain relief, or to address breathing difficulties [6-8]. The new Shim head mask offers complete customization, allowing for discrete adjustments in 0.5 mm increments up to 4 mm, accounting for mask shrinkage post-computed tomography (CT) simulation and providing comfort in cases of post-simulation edema. Additionally, being 50% more rigid than the standard counterpart, the fiberplast Shim mask minimizes positional errors from patient movement and allows for individualized adjustments without removing the thermoplastic mask, promoting patient comfort. This adaptability is advocated to streamline clinical workflows by accommodating potential anatomical changes in patients due to weight fluctuations, thus preventing the need for new mask creations or additional patient simulations [9]. Cone-beam computed tomography (CBCT) imaging facilitates the detection of translational and rotational setup errors. With the robotic couch (hexapod), corrections can be made more accurately, enhancing setup reproducibility [3]. To maintain treatment accuracy, minimizing prolonged treatment times and any delays between imaging and treatment is crucial, as they can introduce positional errors [10]. Incorporating flattening filter-free (FFF) beams in our radiotherapy unit reduces treatment time, while using the hexapod provides precise rotational error corrections before treatment [3]. The primary objective of this study is to determine the immobilization accuracy of the new Shim mask with mouth bite compared to the standard full brain mask in SRT/SRS treatments for brain metastases or tumors. Furthermore, we aim to evaluate the time-efficiency gains with the Shim mask by analyzing the reduction in repeat CBCTs. The following hypotheses were tested: 1. The Integrated Shim™ technology will prove more reproducible than the standard full-head mask; 2. The Integrated Shim™ technology precision will contribute to saving time in the treatment process.

## Materials and methods

Study design and setting

This study utilized a combined retrospective and prospective design to examine the immobilization accuracy of two types of masks used in brain SRT/SRS. All treatments were carried out at the radiotherapy unit and were ethically reviewed and approved by the institutional review board.

Population and sampling

The study included adult participants over 18 years old diagnosed with brain cancer or brain metastases and treated with SRS/SRT. Both male and female patients were included, while pediatric patients were excluded. The retrospective cohort (n=20) consisted of patients previously treated with standard head-only IMRT Reinforced Thermoplastics™, from 2020 to 2022, and the prospective cohort (n = 20) included patients treated with the Innovative Integrated Shim™ technology, from 2022 to 2023.

Patient immobilization, simulation, and treatment planning 

**Figure 1:**
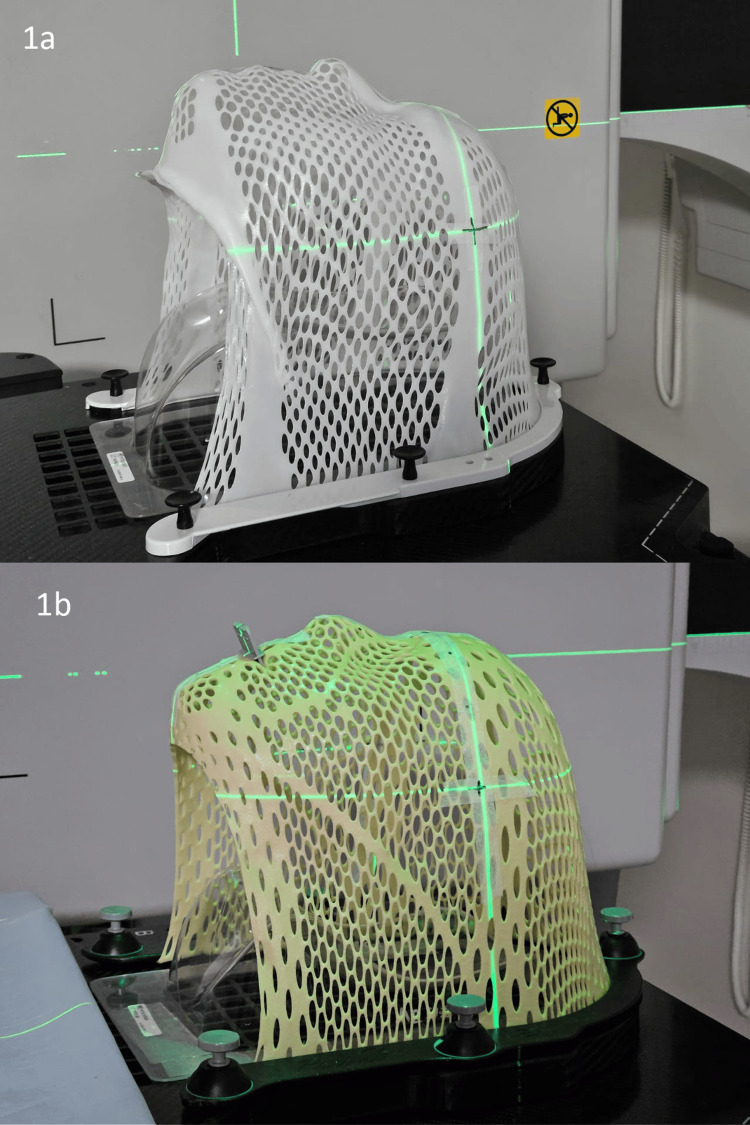
Standard mask without (a) and Shim mask with (b) mouth bite

The retrospective cohort was immobilized using an IMRT-reinforced head-only mask developed by CQ Medica™, formally CVICO RT™ (MTAPUID1824 Style 18, Type-S), that is 2.4 mm thick and composed of a combination of perforated and solid thermoplastic. Patients in the prospective cohort were immobilized using the 3.2 mm Innovative Integrated Shim™ technology also developed by CQ Medical™, formerly Qfix® (RT-A889KYSD, S-frame) and composed of fiberplast head-only mask with an integrated mouth bite inserted through the mask (Figures 1a, 1b; without and with mouth bite).

Both immobilization devices were secured to the treatment couch using an indexer to minimize movement. Patients were positioned supine on the couch in the designated treatment position, aligned using a three-point laser. CT simulation was conducted with a 1 mm slice thickness to encompass the entire head. These scans were then imported into the MONACO planning system. For enhanced delineation of the gross target volume (GTV), MRI fusion, utilizing the MRI neuro navigation protocol, was performed in conjunction with the CT simulation scans. The planned target volume (PTV) included GTV with a 1 mm margin. Treatment fractionation ranged from single to five fractions with variation in the dose prescription.

Treatment procedure

All patients were treated in the same position as the CT simulation, on the Elekta Linear Accelerator with hexapod. Reference marks on the masks were aligned to the in-room lasers (1 mm thickness) and then moved to the treatment isocenter using the shifts provided by the treatment plan. To ensure the accuracy of patient setup, CBCT scans were obtained daily before each treatment session. These scans were automatically registered with the planning CT scan using voxel-based registration to compute the necessary translational and rotational adjustments. The registration occurred within a region of interest "clip box" with bony and soft tissue matching used. Manual adjustments were made at the discretion of the radiation therapist. Images were approved online by the radiation oncologists. The hexapod robotic couch addressed any rotational shifts under 2°. When a setup exhibited shifts more significant than 2°. in the rotational directions, the patient was repositioned as the hexapod in our section is not calibrated to do automatic table correction for greater than 2° due to mechanical limitations. A repeat verification CBCT was performed whenever the patient was repositioned, where translational shifts exceeded 3 mm and rotational shifts exceeded 2°. Note that the data reported were the final offsets, and all shifts were implemented with couch displacement.

Data collection and outcomes

Critical metrics for analysis included absolute shifts in translational directions (anterior, superior, and lateral directions), in cm, and rotational directions (X, Y, and Z) in °. The data reported were the final offset between the best-achieved and planned treatment positions. Other data included patients’ age, gender, SRT/SRS indication (palliative or curative), tumor origin (primary or metastasis), and primary organ of metastasis. Additionally, the number of repeat CBCTs and the time interval between CBCTs were computed and recorded. 

Statistical methods

Data processing and analysis were conducted using the Statistical Package for Social Sciences, Version 21.0 for Windows (SPSS Inc., Chicago, IL, USA). Comparisons between the standard and Shim groups were conducted using the chi-square or Fisher’s exact test for categorical variables and an independent t-test for continuous variables, including absolute values of setup errors in translational and rotational plans. The primary objective was to determine the presence of any significant differences in setup errors between the two groups, both translational and rotational, and subsequently deduce which mask exhibited superior setup reproducibility. The secondary objective is to compare accuracy rates between the two groups in both translational (defined as shift ≤1 mm) and rotational (defined as shift ≤1°) directions; the chi-square test or Fisher’s exact test was used, as applicable. A p-value <0.05 was deemed statistically significant. 

## Results

Participants’ characteristics 

The demographic and clinical characteristics of the participants were well-balanced between the two groups. Most participants were female (75% vs. 80%), received palliative care (70% vs. 75%), and had metastatic tumors (70% vs. 80%) in the standard and Shim groups, respectively (Table 1).

**Table 1:** Participants’ demographic and clinical characteristics Values are frequencies and percentages; except if otherwise specified. Statistically significant difference (p<0.05). Test used: t: independent t-test; F: Fisher’s exact test; otherwise, the chi-square test was used. *Primary tumors (N=10) included meningioma (4), acoustic neuroma (1), astrocytoma (1), cavernous angioma (1), pituitary adenoma (1), and recurrent neurocytoma (1).

Parameter	Standard (N=20)		Shim (N=20)		p-value
Age (mean, SD)	54.45	15.18	48.40	17.53	0.215^t^
Gender					
Male	5	25.0	4	20.0	
Female	15	75.0	16	80.0	1.000^F^
Palliative					
No	6	30.0	5	25.0	
Yes	14	70.0	15	75.0	1.000^F^
Origin					
Primary*	6	30.0	4	20.0	
Metastatic	14	70.0	16	80.0	0.716^F^
Primitive organ					
Breast	11	55.0	12	60.0	
CNS	6	30.0	4	20.0	
Other	3	15.0	4	20.0	0.746

Setup errors

The setup error in standard versus Shim immobilization techniques is presented in Table 2.

**Table 2:** Setup error in standard versus Shim immobilization techniques Values are average absolute setup errors for translational shifts (in mm) and rotational shifts (in °). *Statistically significant difference (p<0.05).

Plan	Direction	Standard (N=20)		Shim (N=20)		p-value
		Mean	SD	Mean	SD	
Translational	Lateral	1.7	1.0	1.0	1.0	0.022*
	Longitudinal	1.7	1.1	1.4	1.2	0.414
	Vertical	1.3	0.8	0.9	0.7	0.180
Rotational	X	0.79	0.43	0.47	0.47	0.030*
	Y	0.77	0.46	0.63	0.53	0.362
	Z	0.77	0.40	0.58	0.49	0.196

The average absolute setup errors for translational shifts (in mm) and rotational shifts (in °) are provided. In terms of translational shifts, there was a statistically significant difference (*p=0.022) observed in the lateral direction, with the standard technique (mean=1.7 mm, SD=1.0) having a higher mean setup error compared to the Shim technique (Mean=1.0 mm, SD=1.0). No significant differences were found in the longitudinal (p=0.414) and vertical (p=0.180) directions. For rotational shifts, a statistically significant difference (*p=0.030) was observed in the X-axis, with the standard technique (mean=0.79°, SD=0.43) having a higher mean setup error compared to the Shim technique (mean=0.47°, SD=0.47). No significant differences existed in the Y-axis (p=0.362) and Z-axis (p=0.196) rotations. 

Accuracy rates in terms of PTV

Accuracy was determined for each fraction by testing the PTV with a cutoff ≤1 mm, meaning all translational shifts were considered accurate within ≤1 mm. Shim was significantly more accurate, at ≤1 mm, in the lateral direction, with an accuracy rate of 70% (70% of the shifts in the lateral direction were ≤1 mm) versus 20% compared to standard techniques (p=0.004). Considering the same accuracy cutoff of ≤1 mm, and although not statistically significant, Shim immobilization showed higher accuracy rates in the longitudinal (50% of the shifts in the longitudinal direction were ≤1 mm vs 35%, p=0.337) and vertical (70% of the shifts in the vertical direction were ≤1 mm vs 40%, p=0.057) directions as well as in the X-axis (90.0% vs 70.0%, p=0.235) (Table 3).

**Table 3:** Comparing accuracy rates in standard versus Shim mobilization techniques Values are accuracy rates for translational shifts (defined as shift ≤1 mm) and in rotational shifts (defined as shift ≤1°). *Statistically significant difference (p<0.05). Test used: F: Fisher’s exact test; otherwise, the chi-square test was used.

Plan	Direction	Standard (N=20)		Shim (N=20)		p-value
		N	%	N	%	
Translational	Lateral	4	20.0	14	70.0	0.004*^F^
	Longitudinal	7	35.0	10	50.0	0.337
	Vertical	8	40.0	14	70.0	0.057
Rotational	X	14	70.0	18	90.0	0.235^F^
	Y	15	75.0	14	70.0	1.000^F^
	Z	16	80.0	16	80.0	1.000^F^

Time-saving

The repeat CBCT scans and the time between the two CBCTs using standard versus Shim immobilization techniques will be displayed in Table 4.

**Table 4:** Repeat CBCT and time between the two CBCTs using standard versus Shim mobilization techniques Total time saving on 20 patients was 145 minutes (2 hours and 25 minutes). CBCT, cone-beam computed tomography

Parameter	Standard (N=20)		Shim (N=20)	
	N	%	N	%
Repeat CBCT fraction 1	12	60.0	0	0.0
Repeat CBCT fraction 2	2	10.0	0	0.0
Total repeat CBCT	14	70.0	0	0.0
	Mean	SD	Mean	SD
Time between CBCTs (minutes)	10.4	4.5	-	-

In the standard group (N=20), 70% of the participants (N=14) required repeat CBCT scans, with the majority being on the first treatment fraction (60%). In contrast, none of the participants in the Shim group (N=20) were required to repeat CBCT scans, indicating a significant difference between the two groups. 

The mean time between CBCT scans in the standard group was 10.4 minutes (SD=4.5). Overall, using the Shim immobilization technique resulted in a total time saving of 145 minutes (2 hours and 25 minutes) for the 20 patients included in the study. 

## Discussion

Summary of findings

Meticulous attention to immobilization details, as per the ESTRO ACROP guidelines, is integral to enhancing the precision and effectiveness of radiotherapy [11]. This retrospective-prospective study aimed to compare the setup errors and time savings associated with the use of the Shim immobilization technique concerning the standard immobilization technique. We applied the two immobilization techniques in two cohorts of patients who showed comparable demographic and clinical characteristics. The Shim technique showed significantly lower errors in lateral translational and X-axis rotational shifts than the standard technique. This was supported by a substantially higher accuracy rate at the lateral direction among the Shim group, considering a shift cutoff ≤1 mm and a better overall performance in the other directions without statistical significance. Notably, the Shim technique eliminated the need for repeat CBCT scans in all participants. These results highlight the benefits of the Shim technique in reducing setup errors and saving time during treatment.

Importance of an efficient immobilization

Immobilization accuracy is vital in SRS and SRT treatments for brain metastases or tumors. Given the high radiation dose these techniques deliver per session, the associated peripheral doses could lead to significant long-term complications [12]. Therefore, efficient immobilization ensures that radiation is accurately targeted to the tumor, maximizing therapeutic efficacy, and minimizing exposure to surrounding healthy brain tissue. This precision is essential for therapeutic success, preservation of neurological function, and reduction of complications such as radiation-induced damage. Proper immobilization also ensures that the designed treatment plan based on imaging studies is meticulously executed during each session [12-14].

Immobilization devices, critically indexed and secured to the couch, restrict translational and rotational errors. Any discrepancies in positioning, even due to inadequacies in neck rests, can affect the treatment's accuracy and efficacy, mainly in brain procedures [11]. Thus, meticulous attention to immobilization is pivotal for optimized therapeutic outcomes.

Accuracy and reproducibility of Shim mask

The current comparative study demonstrated that the Shim mask technique decreased the setup error by an average of 0.7 mm laterally and 0.32° in the X-axis. Furthermore, while not reaching statistical significance, there was a reduction in the mean setup errors across all other plans and directions within the Shim group. The Shim mask performed better than the standard immobilization technique by considering cutoff points of 1 mm in translational and 1° in rotational directions. The difference in performance in the lateral direction was notable, as 70% of the Shim group patients were within the defined cutoff versus only 20% of the control group. The need for a repeat CBCT in the standard mask can be attributed to different factors including the composition of the mask (2.4 mm thermoplast), which is not as sturdy as the Shim mask (3.2 mm fiberplast), absence of a mouth bite in the standard group also plays a pivotal role in immobilization and treatment reproducibility, and the limitation of the hexapod in our section to do automatic table correction for greater than 2°. This difference probably explains the substantial time saving resulting from a lesser need for repeat CBCT in the Shim group. 

Historically, SRS has been conducted using an invasive fixed head ring to determine the stereotactic coordinates of the target, ensuring immobilization and positioning accuracy of less than 1 mm during imaging and treatment. However, various frameless stereotactic systems have recently been introduced as alternatives to these invasive fixation techniques. These frameless systems have reported positioning accuracies ranging from 1 mm to 4 mm. For example, a study analyzing the accuracy of the TALON system showed exceptional precision for single-fraction SRS with a mean magnitude of isocenter translation of 0.99 mm, which slightly deteriorated to 1.38 mm over a six-week fractionated stereotactic treatment course [15]. The Shim technique, as presented in our study, demonstrated a comparable mean setup error of 1.0 mm and 0.9 mm, in the lateral and vertical direction, respectively. 

Regarding rotational errors, the Shim group demonstrated a mean rotation of less than 1° in all three rotational directions upon initial setup, consistently lower than that observed in the standard group. Moreover, by considering a cutoff ≤1°, the Shim mask showed superior performance in the X-axis, compared to the standard group, although this was not statistically significant. A study by Roper et al. showed that the target coverage deteriorated when rotational error increased to 2°. The dose of 95% of the PTV (D95) and the volume covered by 95% of the prescribed dose (V95) were <90% in about 20% of the cases [16]. Another significant clinical concern is the potential for rotational errors to result in an overdose of normal tissues. 

These comparative results suggest the superior performance of the Shim mask, which can lead to better patient outcomes, as the likelihood of damage to critical brain structures is reduced [17,18]. This is crucial given the brain's sensitivity to radiation and the potential for long-term neurological sequelae from off-target radiation. Furthermore, it has been evidenced that non-brain-directed radiation therapy can induce bystander brain damage, marked by hypometabolism and neuroinflammation, suggesting potential cognitive impairments in treated cancer patients [18].

Timesaving and workflow efficiency

Timesaving in the context of SRS and SRT treatments for brain metastases or tumors is not just a logistical advantage but has excellent clinical implications. Improved setup, as evidenced by the reduced need for repeat CBCT, directly translates to a more efficient treatment workflow. In this study, using the Shim mask eliminated the need for repeat CBCT in all patients, whereas 14 out of 20 patients treated with the standard technique required a repeat CBCT. This led to an average time saving of 10 minutes per patient. Such time saving is critical, as reducing the time a patient spends in the treatment and CT simulation suite can enhance patient comfort and reduce anxiety, which is particularly important given the challenging nature of treating brain tumors and metastases [19,20]. Although patient comfort was not addressed in this study, none of the patients complained that the Shim mask was tight.

On the other hand, efficient workflow can potentially allow for the treatment of more patients, optimizing resource utilization. The associated cost savings, despite not being assessed in this study, can have significant implications for healthcare budgeting and resource allocation. It is worth noting that the costs of repeat CBCT in radiation therapy, especially for brain tumor treatment, are substantial, in addition to the inherent expenses of SRS/SRT treatment [21]. The peripheral dose from CBCT, in addition to the in-field dose, plays a crucial role in the long-term care of radiation oncology patients, emphasizing the importance of considering these doses when evaluating the overall radiation exposure to the patient [22].

Limitations

While the results provide valuable insights into the immobilization accuracy of the two compared techniques, it is necessary to recognize this study's potential biases and limitations. The study was conducted within a single institutional setting, which restricts the generalizability of the findings to broader clinical contexts and other institutions with potentially varying standards and practices. Additionally, this study did not include the intrafraction patient motion, and with limited resources, the only option was to do CBCT at the end of the treatment. However, this is at one point and does not account for patient motion during treatment. Future multicenter studies with larger sample sizes would further substantiate and expand upon these findings. 

## Conclusions

Accurate immobilization in SRT for brain tumors is crucial for effective treatment and patient safety. This study highlights the superior performance of the Shim immobilization technique compared to the traditional standard mask. The Shim technique reduced setup errors and improved accuracy, resulting in significant time savings by eliminating the need for repeat CBCT scans. As radiotherapy facilities aim to enhance patient outcomes, comfort, and workflow, the findings of this research suggest that the Shim technique could be a valuable tool for patient immobilization and treatment precision, especially in the absence of surface-guided radiotherapy.

## References

[1] Zhang M, Zhang Q, Gan H, Li S, Zhou SM (2016). Setup uncertainties in linear accelerator based stereotactic radiosurgery and a derivation of the corresponding setup margin for treatment planning. Phys Med.

[2] Tomihara J, Takatsu J, Sugimoto S, Shikama N, Sasai K (2021). Intrafraction stability using full head mask for brain stereotactic radiotherapy. J Appl Clin Med Phys.

[3] Hartgerink D, Swinnen A, Roberge D (2019). LINAC based stereotactic radiosurgery for multiple brain metastases: guidance for clinical implementation. Acta Oncol.

[4] Kirkpatrick JP, Wang Z, Sampson JH (2015). Defining the optimal planning target volume in image-guided stereotactic radiosurgery of brain metastases: results of a randomized trial. Int J Radiat Oncol Biol Phys.

[5] Lightstone AW, Tsao M, Baran PS (2012). Cone beam CT (CBCT) evaluation of inter- and intra-fraction motion for patients undergoing brain radiotherapy immobilized using a commercial thermoplastic mask on a robotic couch. Technol Cancer Res Treat.

[6] Mullaney T, Pettersson H, Nyholm T, Stolterman E (2012). Thinking beyond the cure: a case for human-centered design in cancer care. Int J Des.

[7] Clover K, Oultram S, Adams C, Cross L, Findlay N, Ponman L (2011). Disruption to radiation therapy sessions due to anxiety among patients receiving radiation therapy to the head and neck area can be predicted using patient self-report measures. Psychooncology.

[8] Mulla Z, Alwassia RK, Senan EM (2020). A comparative study between open-face and closed-face masks for head and neck cancer (HNC) in radiation therapy. Rep Pract Oncol Radiother.

[9] (2023). Oncology Imaging Systems. Integrated Shim System. https://www.oncologyimaging.com/products/radiotherapy/Head-and-Neck/Integrated-Shim-System.

[10] Tarnavski N, Engelholm SA, Af Rosenschold PM (2016). Fast intra-fractional image-guidance with 6D positioning correction reduces delivery uncertainty for stereotactic radiosurgery and radiotherapy. J Radiosurgery SBRT.

[11] Leech M, Coffey M, Mast M, Moura F, Osztavics A, Pasini D, Vaandering A (2017). ESTRO ACROP guidelines for positioning, immobilisation and position verification of head and neck patients for radiation therapists. Tech Innov Patient Support Radiat Oncol.

[12] Fujimoto D, von Eyben R, Gibbs IC (2018). Imaging changes over 18 months following stereotactic radiosurgery for brain metastases: both late radiation necrosis and tumor progression can occur. J Neurooncol.

[13] Chang WS, Kim HY, Chang JW, Park YG, Chang JH (2010). Analysis of radiosurgical results in patients with brain metastases according to the number of brain lesions: is stereotactic radiosurgery effective for multiple brain metastases?. J Neurosurg.

[14] Nath SK, Lawson JD, Wang JZ (2010). Optically-guided frameless linac-based radiosurgery for brain metastases: clinical experience. J Neurooncol.

[15] Salter BJ, Fuss M, Vollmer DG (2001). The talon removable head frame system for stereotactic radiosurgery/radiotherapy: measurement of the repositioning accuracy. Int J Radiat Oncol.

[16] Roper J, Chanyavanich V, Betzel G, Switchenko J, Dhabaan A (2015). Single-isocenter multiple-target stereotactic radiosurgery: risk of compromised coverage. Int J Radiat Oncol Biol Phys.

[17] Lamba M, Breneman JC, Warnick RE (2009). Evaluation of image-guided positioning for frameless intracranial radiosurgery. Int J Radiat Oncol Biol Phys.

[18] Chang HH, Lee HF, Sung CC, Liao TI, Huang YJ (2013). A phantom study of the immobilization and the indications for using virtual isocenter in stereoscopic X-ray image guidance system referring to position localizer in frameless radiosurgery. J Appl Clin Med Phys.

[19] Burns M, Campbell R, French S, Dhillon HM, Butow PN, Pritchard A, Sundaresan P (2022). Trajectory of anxiety related to radiation therapy mask immobilization and treatment delivery in head and neck cancer and radiation therapists’ ability to detect this anxiety. Adv Radiat Oncol.

[20] Oultram S, Findlay N, Clover K, Cross L, Ponman L, Adams C (2012). A comparison between patient self-report and radiation therapists’ ability to identify anxiety and distress in head and neck cancer patients requiring immobilization for radiation therapy. J Radiother Pract.

[21] Rahman F, Seung SJ, Cheng SY, Saherawala H, Earle CC, Mittmann N (2016). Radiation costing methods: a systematic review. Curr Oncol.

[22] Perks JR, Lehmann J, Chen AM, Yang CC, Stern RL, Purdy JA (2008). Comparison of peripheral dose from image-guided radiation therapy (IGRT) using kV cone beam CT to intensity-modulated radiation therapy (IMRT). Radiother Oncol.

